# Climate Change and Malaria in Canada: A Systems Approach

**DOI:** 10.1155/2009/385487

**Published:** 2009-01-04

**Authors:** L. Berrang-Ford, J. D. MacLean, Theresa W. Gyorkos, J. D. Ford, N. H. Ogden

**Affiliations:** ^1^Department of Geography, McGill University, 805 Sherbrooke Street West, Montreal, QC, Canada H3A 2K6; ^2^McGill University Centre for Tropical Diseases, Montreal General Hospital, Department of Medicine, McGill University, Montreal, QC, Canada H3G 1A4; ^3^Division of Clinical Epidemiology, McGill University Health Centre, Royal Victoria Hospital, V Building, 687 Pine Avenue West, Montreal, QC, Canada H3A 1A1; ^4^Department of Epidemiology, Biostatistics and Occupational Health, McGill University, Montreal, QC, Canada H3A 1A2; ^5^Public Health Agency of Canada and Faculté de médecine vétérinaire, Université de Montréal, CP 5000, Saint Hyacinthe, QC, Canada J2S 7C6

## Abstract

This article examines the potential for changes in imported and autochthonous malaria incidence in Canada as a consequence of climate change. Drawing on a systems framework, we qualitatively characterize and assess the potential direct and indirect impact of climate change on malaria in Canada within the context of other concurrent ecological and social trends. Competent malaria vectors currently exist in southern Canada, including within this range several major urban centres, and conditions here have historically supported endemic malaria transmission. Climate change will increase the occurrence of temperature conditions suitable for malaria transmission in Canada, which, combined with trends in international travel, immigration, drug resistance, and inexperience in both clinical and laboratory diagnosis, may increase malaria incidence in Canada and permit sporadic autochthonous cases. This conclusion challenges the general assumption of negligible malaria risk in Canada with climate change.

## 1. Introduction

Canada's climate could support, and has supported, local malaria transmission in the past. Malaria was introduced to continental North America in the 16th and 17th centuries by European colonists and African slaves [1]. The disease spread with settlement until being controlled in the early 1900s and eradicated in the 1950s [1]. Extensive debate and research has focused on the potential for climate change to alter or increase malaria distributions and incidence both globally and regionally. Global models and research discourse focuses—justifiably—on the impact of climate change on the spread and magnitude of global endemic malaria in at-risk regions and regions at the margins of current endemic distributions. Changes in malaria incidence in Canada and similar northern countries are assumed to be negligible. In this paper we challenge this assumption.

The 4th assessment report (FAR) of the Intergovernmental Panel on Climate Change (IPCC) highlights, with increased confidence, the projected impact of climatic change on infectious diseases and human health [2]. These projections are supported by a growing body of literature which has contributed to the characterization and quantification of climate as a determinant of disease distributions and incidence [3–10]. The assessment of climate impacts on infectious diseases, however, is challenged by often complex interactions of climatic determinants with other ecological, economic, and social determinants of disease incidence [5, 6, 9, 10]. This has led to efforts to quantify the disease burden that is specifically attributable to climate change [4, 8], but disentangling the causal contribution of anthropogenic climate change from complex disease systems poses both analytical and conceptual difficulties. Analytically, it is often difficult to characterize and quantify causes of disease variation in time and space, and to quantify these while controlling for variation in nonclimatic determinants. Furthermore, both the direct and indirect effects of climate change are likely to impact vector-borne disease occurrence; for example, extreme weather events (or indeed long-term changes in climate) may result in population migration and subsequent changes in population health and exposure. Increasing sophistication in transmission and systems modeling, however, has provided innovative approaches to confront this challenge [5, 7, 9]. The IPCC FAR, for example, does itself integrate social and ecological determinants of disease [3].

Within the climate change and health literature, there have been parallel developments in the application and development of systems frameworks for assessing environmental change impacts on infectious disease [6, 10–13]. These approaches arise from a range of (inter)disciplinary and pedagogical roots, including environmental health, social epidemiology, environmental change, and systems theory [13]; the emergence of conceptual systems frameworks has included eco-epidemiology [14], ecohealth [15, 16], social epidemiology [17], and vulnerability science [18, 19]. Ecosystem health approaches, for example, have been widely used to provide new and integrative frameworks for conceptualizing, understanding, and characterizing complex dynamics within health systems [6, 20–23]. These approaches draw on general and complex systems theory [24–27] to conceptualize health problems as highly integrative systems affected by interacting processes of social and ecological complexity. The theoretical basis of these approaches is that understanding complex systems can only be achieved by looking at how different parts of a system interact together rather than from teasing them apart [28]. Climate change, in this context, is one of several determinants of infectious disease occurrence, whose impact is superimposed upon, and moderated by, parallel changes in nonclimatic determinants. The utility of these frameworks is thus not necessarily in isolating the attributable burden of disease due to climate change, but rather in explicitly characterizing the *cumulative* or *integrative* impact of climate change within the context of changes in other disease determinants. Such frameworks are particularly useful for preliminary characterization and identification of key processes, interactions, scales, and feedbacks—an exercise which can inform and guide integrative attribution modeling [12, 13, 20].

In this paper, we use a systems approach to begin to assess how climate change, by having multiple proximal and distal influences on determinants of transmission, might affect the occurrence of malaria in Canada. It has been generally concluded that there is negligible risk of malaria in Canada and similar northern countries due to climate change [27–29]. This assessment has largely been based on the assumption that the importance of malaria in these countries rests solely on the likelihood of endemic malaria becoming established. In this paper, we challenge this assumption, drawing on four key premises. (1) Absence of risk of *endemic* malaria does not preclude the potential for changes in incidence due to imported or sporadic autochthonous (i.e., locally acquired) malaria. (2) Even small changes in malarial incidence or emergence of autochthonous cases in Canada and other nonendemic countries could have important implications for public health systems. (3) Current models of climate impacts on malaria are not designed to effectively or accurately evaluate the potential for changing malaria incidence in peripheral regions and should not be interpreted as such. (4) Systems approaches provide a useful framework for conceptually and methodologically integrating social and biophysical determinants of disease.

We begin the paper by describing the methods used in the study, before reviewing the nature of malaria incidence in Canada. We then assess how climate-malaria links have been approached in the literature in general and argue that assumptions of limited risk in Canada are not supported by existing climate-malaria research, and merit re-examination in the context of sporadic incidence and the role of nonclimatic determinants. The paper then explores how climate change might affect malaria incidence in Canada based on an understanding of the malaria transmission cycle and its climatic determinants. Using a systems approach, we assess how nonclimatic determinants of malaria risk may exacerbate or moderate climate-related changes in malaria incidence. The characterization presented here represents a preliminary qualitative review and synthesis of existing knowledge and literature and should therefore be considered descriptive and exploratory.

## 2. Methods and Approach

A summary of common themes and considerations used in systems frameworks was developed to guide this review (Table 1). A systems graphic was developed by adapting the malaria life cycle model to identify and include both proximal and distal transmission determinants. Key parameters were reviewed and assessed with respect to trends, interactions, and potential impact on transmission. We performed an integrative review and analysis of existing literature and data related to malaria transmission and dynamics, Canadian malariology research, climate change science, projections for Canadian climate, demographic trends, international travel, and current models and research related to both global and regional impacts of climate change on malaria transmission.

Canadian malaria data were acquired from the Public Health Agency of Canada Notifiable Diseases registry [29]. Distributions of mosquito vectors were reviewed to identify competent vectors with distributions in Canada and with the highest vector potential for parasite transmission [1, 30–37]. Existing records of autochthonous malaria cases were reviewed, recorded, and combined to map autochthonous malaria in Canada and the United States (1957–2003) [1, 38–45]. These cases were overlaid with key vector distributions using ArcMap (ArcInfo 9.2, Environmental Systems Research Institute, Redlands, Calif, USA).

Temperature data were compared to parasite development thresholds and dynamics using three sources of data: effects of temperature on the sporogonic cycle of *Plasmodium* spp. in mosquitoes, Canadian climate data, and downscaled climate change projections. The effects of temperature on the duration of development of the parasites in the mosquito were as follows: *P. vivax* requires approximately 30 days at 18°C or 20 days at 20°C, while *P. falciparum* requires approximately 30 days at 20°C. Above 33°C or below 16/18°C (for *P. vivax* and *P. falciparum*, resp.), the cycle cannot be completed and transmission cannot occur [1, 46]. Data on the maximum number of consecutive days >18°C in Toronto (1970–2006) were calculated using archived Environment Canada climate data [46]. Data from Toronto were selected since Toronto is the largest Canadian city within the distributional range of a competent malaria vector. Downscaled climate change projection data were used to characterize projected temperature changes on transmission potential. The climate change projections were obtained from interpolation (for Chatham, Ontario) of output from the CGCM2 (Canadian Coupled Global Climate Model 2) [47] that incorporated estimated forcing calculated in emission scenario forcing “A2” [48]. The output was downscaled using LARS-WG stochastic weather generator. LARS-WG was calibrated with 30 years of daily weather observations at Chatham (and its predecessors) obtained from the Environment Canada database. Chatham was selected because it is the location closest to Toronto for which we already have downscaled projections. The data and methodology used here are described in detail by Ogden et al. [49].

## 3. Malaria in Canada and USA

As recently as the 1820–30s, Canada experienced malaria epidemics, including severe outbreaks during construction of the Rideau Canal in eastern Ontario, and outbreaks in Montreal and the prairies [50]. Records suggest endemic malaria occurred in the mid to late 1800s on the shores of Lake Ontario from Kingston to Hamilton, along the northern shore of Lake Erie, along the whole St. Lawrence River and its tributaries, in parts of the western provinces, and sporadically in Quebec City and Halifax [36]. Incidence declined steadily in the early 1900s, and its eradication is attributed to increased urbanization and improved socioeconomic conditions which decreased mosquito populations, decreased human contact with mosquitoes, and improved the speed and effectiveness of case treatment [36, 51]. While current socioeconomic conditions have dramatically reduced the risk of local malaria transmission, historical incidence supports the potential for autochthonous malaria in Canada under conditions favouring transmission. For this reason, we evaluate the potential impact of shifts in the climatic and nonclimatic determinants of malaria on the balance of transmission potential.

For malaria transmission to occur, three key factors need to coincide: competent vectors, a suitable climate for transmission cycles (i.e., completion of parasite development in the vector), and infected, infective humans. The first two factors are present in some locations in Canada, and travel and immigration could potentially introduce parasites into local human populations. However, in recent decades these factors have not been sustained at levels sufficiently high or for periods sufficiently long, for transmission cycles to be established, even at a local level. This is because (1) the numbers of infected and infective humans have been too low (due to high standards of living and ready access to medical services and antimalarial chemotherapy) and (2) vector abundance and rates at which mosquitoes could bite humans have not been sufficiently high (due to a combination of climate, water management, housing conditions, and mosquito control). Thus, while malaria transmission occurred in Canada in the 19th century and the requirements for transmission of malaria theoretically exist in Canada, transmission potential since 1900 has been too low to permit local transmission [36, 52–54]. Changes in these and other factors, directly or indirectly associated with climate change, however, have the potential to affect this balance.

An average of over 500 travel-related cases are reported annually in Canada (1989–2004, Figure 1; [29, 55]). This number, which likely represents fewer than half of actual cases [55], is comparable to the incidence of West Nile Virus in Canada in most years (annual average of 459 clinical cases up to 2006 [56])—a disease which has generated widespread media and public health attention in Canada. While the United States records far more cases ( >1000/year [57]), reported malaria incidence per capita in Canada is at least three times higher than its southern neighbour; the reasons for this difference remain unclear [55, 58]. Nearly, all malaria cases in North America are imported cases brought into the country by people who have become infected while visiting, or after arriving from, an endemic country [59]. Travel and immigration were also associated with two of Canada's most significant recent outbreaks. The first, in 1995–97, involved Canadians traveling to the Indian Punjab region, which was experiencing a *P. vivax* outbreak at that time [59]. The second involved an outbreak in Quebec in 2001-2002 in a population of central and east African refugees recently arrived in Canada from Tanzanian refugee camps [58–60]. While there are few malaria deaths in Canada each year, all malaria cases raise significant community and public health concern [55].

**Figure 2:**
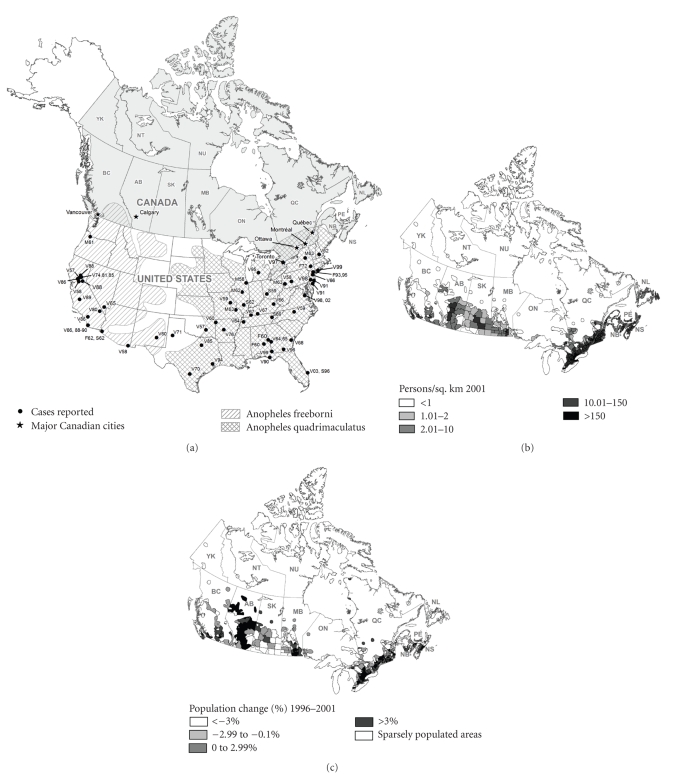
Geographic distribution of vectors of malaria, cases of local mosquito-borne transmission during the period 1957–2003 Figure 2(a), population density Figure 2(b), and population change Figure 2(c) in Canada. (a) shows the geographic distribution of vectors of malaria and cases of local mosquito-borne transmission during the period 1957–2003. Black dots represent location of cases of malaria in the United States and Canada presumed to be acquired from local mosquito-borne transmission between 1957 and 2003 (Source: [1, 38–45]. Each dot represents one or a cluster of cases in a given year. Labels include species type (V = *P. vivax,* F = *P. falciparum*, M = *P. malariae*, S = species unknown) and date. Locations are approximate. Hashed areas represent the approximate distributions of the two most important competent malaria vectors in Canada. (Sources of malaria data: [1, 30–34]). See Table 2 for full names of Canadian provinces. (b) and (c): population density (2001) and population change (1996–2001) in Canada. Source: Population Ecumene Census 2001, GeoGratis, Natural Resources Canada.

While most cases of malaria in the United States are imported, there have been a number of cases of autocthonous malaria, whereby people with no history of travel to endemic areas have become infected by locally infected mosquitoes. Between 1957 and 2003, there were 156 cases of locally transmitted malaria in US [40]. These cases have not been confined to the southern United States (Figure 2(a)); locally-transmitted cases, for example, have been reported in Virginia, New York, and Michigan [1, 40]. Locally acquired outbreaks are often reported near urban centres or airports, where large influxes of travelers and immigrants are found [40]. Increased air travel, increasing drug resistance, and changing environmental conditions have raised concern over malaria re-emergence in the United States [1]. In Canada, one case of suspected locally-acquired autochthonous malaria was reported in a Toronto woman in 1997 [38, 61] (Figure 2(a)). An unexplained death due to *falciparum* malaria was also recorded in Quebec in 1974, raising questions of local transmission, although this is unlikely give that local climate conditions at that time could not have supported transmission [61, 62].

In Canada, there are six species of *Anopheles* mosquito, of which only two are potentially important vectors of malaria (Table 2). *An. quadrimaculatus*, found in southern Ontario and Quebec, and *An. freeborni* which occurs in south-western British Columbia (BC) (Figure 2(a), Table 2) are considered to be the most important vectors for potential autochthonous transmission in the northern United States, and by extension, Canada [1, 30, 32–34]. Although the regions where these two mosquito species occur represent only a small proportion of Canada's territory, they are located in areas where at least half of Canada's population reside and where the majority of population growth in Canada is occurring [62] (Figures 2(b)-2(c)). The potential population at risk could increase with even small expansions in the geographic ranges of the vectors themselves, and of the areas climatically suitable for parasite transmission. Increased mosquito abundance, duration of seasonal survival, and parasite replication *within existing distribution ranges*, however, would likely affect population risk more than range expansion. There is little information on local or urban mosquito abundance and biting within these ranges; existing mosquito distribution data for Canada (Figure 2(a)) are based on rough estimates dating to the 1970s, with much of the available data more than several decades old [30, 31, 35, 37]. Current, updated, and detailed distributions of competent malaria vectors in Canada remain unknown.

## 4. Climate Change and Global Malaria

Climatic factors are important determinants of malaria transmission. The parasite can only be transmitted from a mosquito to a human once it has completed a complex cycle of development and multiplication inside the mosquito, called the sporogonic cycle [1]. The length of this cycle (often called the extrinsic incubation period) depends on the parasite species and ambient temperature [1]. Therefore, for mosquitoes to transmit infection from an infected human to an uninfected human, ambient temperatures must be sufficiently high, for a sufficiently prolonged period, (1) for mosquitoes to acquire infection by biting an infected human, (2) for parasites to develop in the mosquito, and then, (3) for the mosquito to bite another human and transmit the parasites. The lifespan of the mosquito is related, among other factors, to air temperature, humidity, and rainfall, which also affect mosquito abundance and the rate at which mosquitoes bite humans [1, 50, 51, 63]. Climate has, therefore, multiple effects on malaria transmission.

Malaria periodicity and outbreaks have long been recognized to be associated with climate and climate fluctuations, particularly, on the fringes of global malaria distribution [64–67]. Global malaria outbreaks have been regularly linked, for example, to heavy rains associated with El-Nino events [64–66]. In the United States, hotter and more humid weather conditions were a common factor in local outbreaks of malaria, including a case at a Michigan campsite in 1995 [1, 68]. These warmer, wetter conditions can increase the survival of the mosquito and reduce the required length of the sporogonic cycle sufficiently to allow the parasite to develop and the mosquito to become infectious where it otherwise would not. Similar localized outbreaks in nonendemic northern countries have also been associated with particularly warm weather [69].

Predictions of global climate change [70] have lead to extensive research interest into its potential impact on malaria incidence [32, 71–79], but how climate change may affect the incidence and distribution of malaria is much debated [80–82]. Differing opinions generally arise from differing conceptual and methodological approaches to malaria modeling. Some research in this area has predicted significant global or regional spread based on biological models that incorporate some climate-driven variables that directly affect the basic reproductive number of malaria (*R*_0_) (particularly parasite replication in the vector); these reflect predictions of extensions in transmission season and geographic range, where there may be a potential for transmission cycles to occur [83, 84]. These models, which focus on transmission *potential,* can, however, over-predict both the impact of climate and current disease distributions. More conservative projections based on statistical approaches, such as that by Rogers and Randolph [85] have suggested negligible change in global malaria distributions. These are based on *current* global distributions and statistical estimates of existing incidence risk. It is difficult to explicitly incorporate nonclimatic factors such as health care, local habitat, and vector control into global malaria models; these determinants are therefore generally absent from global projections, though efforts have been made to integrate and reflect socioeconomic vulnerability and adaptive capacity into global scenarios in a general sense [9]. Rogers and Randolph, for example, acknowledge this challenge conceding that the model predictions are less reliable in marginal areas, where mosquito life-spans barely exceed incubation periods for the parasite, and acknowledging that nonclimatic factors are particularly important in determining the balance of transmission in these areas. Van Lieshout et al. [9] note that more accurate integration of socioeconomic variables in malaria modeling will require research at regional and national scales.

Research has been conducted on the potential for climate impacts on malaria in northern countries, particularly, the UK. Kuhn et al. [86], in an analysis of the risk of malaria re-emergence in Britain, concluded that despite an increase in the transmission potential due to climate change, the importance of nonclimatic factors (including medical systems, socioeconomic conditions, and agricultural changes) are likely to prevent emergence of endemicity. This example raises a second important point: the focus of climate-malaria models and assessments on distributions and spread of *endemic* malaria. While this is certainly justified in the prioritization of global health priorities and infectious disease burden, it does not negate the potential for climate impacts on sporadic autochthonous or imported cases in marginal or peripheral regions. Given the importance of nonclimatic factors on malaria transmission in marginal regions such as Canada, and using an assumption that nonendemic malaria incidence is of research and public health relevance, global climate-malaria models cannot be used to infer risk in such regions. That is to say that while existing models are rigorously developed and valuable to global climate-malaria projections, these models are not designed to predict changing risk in nonendemic areas, where climatic conditions for transmission are marginal.

## 5. Climate Change and Malaria in Canada

In the case of Canada and other developed regions on the periphery of the climatic range of malaria transmission, nonclimatic and local factors are important determinants affecting the balance of transmission potential. For these regions, with our current information, it is difficult to quantify whether increased climatic suitability would be sufficient to push the probability of malaria transmission beyond the threshold at which current localized and social factors become insufficient to inhibit transmission. Given Canada's northern climate and well-established social, health and economic systems, Canada is at negligible risk of experiencing endemic or regular malaria transmission despite the presence of competent *Anopheles* vectors [32, 87]. The questions of climate change impacts on malaria risk in Canada are not so much whether Canada will become an endemically infected country, but more whether changing climate determinants will have an impact on current incidence, and whether we can expect to see cases of locally-acquired autochthonous transmission in Canada. The answers to these questions require simultaneous evaluation of potential climatic effects as well as trends in nonclimatic determinants of malaria transmission.

Predictions of shorter winters and increasing spring and summer temperatures, including prolonged summer heat waves [88, 89], could promote mosquito abundance and parasite replication in the summer in Quebec, Ontario, and British Columbia. Conversely, predictions of decreased summer rainfall, particularly in southern BC where there are already summer rainfall deficits [88, 89], could reduce mosquito survival. Southern Quebec and Ontario already have hot, humid summers with extended periods of high temperatures. Hotter summers, which are predicted by climate change in this region [88, 89], may result in a decrease in the number of consecutive warm days required for the parasite to develop within the mosquito and for the mosquito to become infectious. Additionally, milder winters and spring increases in precipitation in Ontario and Quebec [88, 89] may promote early mosquito abundance or increased winter survival of infective mosquitoes. These represent presumed potential impacts, though a number of descriptive reviews and qualitative assessments—particularly related to West Nile Virus—have suggested that climate changes will affect mosquito abundance and distributions in Canada [54, 87, 90–93], no quantitative models or results have yet been published to more certainly explore the potential impact of climate change on Canadian mosquito vectors.

**Figure 3:**
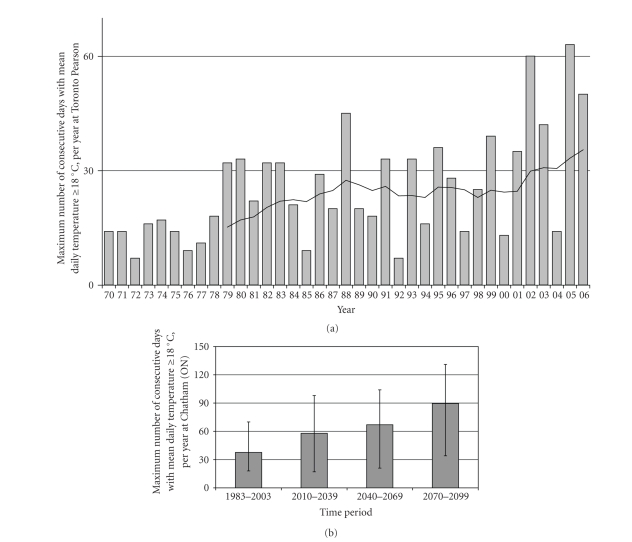
(a) Annual number of consecutive days ≥18°C, Toronto. Bars indicate the number of consecutive days per year that temperatures ≥18°C. A trendline (solid line) shows the 10-year moving average for the data. The trendline suggests that in the last few years, we have begun to experience sufficiently prolonged summer warm periods to support parasite replication and malaria transmission potential. In 2002 and 2005, the number of days above 18°C was sufficient to support 2 cycles of *P. vivax* replication. These data should be considered conservative since each degree-day ≥18°C will reduce the remaining time required for parasite replication. Additionally, breaks in consecutive warm days ≥18°C do not necessarily prohibit continued development once temperatures rise [35]. Source of climate data: Environment Canada [46]. (b) Annual number of consecutive days ≥18°C projected for 2010–2099, Chatham (ON). Bars indicate the number of consecutive days per year that temperatures are projected to reach or exceed 18°C. Error bars indicate the range of values during each time period. The climate change projections were obtained from interpolation (for Chatham, Ontario) of output from the CGCM2 (Canadian Coupled Global Climate Model 2) [47] that were downscaled using LARS-WG stochastic weather generator. LARS-WG was calibrated with 30 years of daily weather observations at Chatham (and its predecessors) obtained from the Environment Canada database. The output used here was obtained using emissions scenario A2 (business as usual). The data and methodology used here are the same as described in Ogden et al. [49]. The projected trend shown here indicates increasingly extended summer warm periods sufficient to support multiple parasite replication cycles.

In recent decades, there have been several years when Toronto has experienced more than 30 consecutive days above 18°C (Figure 3(a)), conditions potentially supporting *P. vivax* development in the vector. In 2002 and 2005, there were sufficient warm days to allow for two full replication cycles. The actual time required for parasite development depends upon an accumulation of “degree-days,” a count of the cumulative number of days when temperatures exceed the minimum threshold for development, with each degree above the threshold contributing to an additional degree-day [1, 49, 82]. These days do not necessarily need to be consecutive; some parasite species can survive temperatures below and above the minimum threshold and continue development once temperatures rise again [35], although these relationships are poorly understood, particularly in northern latitudes.

Projected temperatures for Chatham in southwestern Ontario [47, 49] suggest a doubling of the summer period capable of supporting parasite development (based on a period of 30 days over 18°C) within the next 50–75 years (Figure 3(b)). Given that many of these days are well over 18°C and the number of days required for replication decreases at higher temperatures, these estimates can be considered conservative. This trend may be generalizable within the southern Ontario region, and while the degree of warming predicted varies among climate models and emissions scenarios, all model predictions in the IPCC FAR [2] and the Canadian National Impacts Assessment [94] indicate a warming trend in the Canadian regions where competent malaria vectors currently exist.

These projections can be placed within the context of global malaria modeling. Van Lieshout et al. [9], for example, suggest that regions where the transmission season increases from 0 to 1 or 2 months per year may experience large increases in population at risk. Canada fits within this range, and may experience increases of up to 3 months per year (Figure 3(b)). Van Lieshout et al. [9] also note that this transmission will be unstable (or sporadic) and that absolute risk remains low. Within the context of a national public health system such as Canada's, however, even low risk of emerging sporadic malaria is of importance for public health services and programming.

Changes in the variation and extremes of rainfall and temperature may be as important as trends in average temperature conditions for transmission potential. Southern Quebec is projected to experience both increases in average summer rainfall as well as more frequent and extreme rainfall events. Despite predictions of reduced summer rainfall in southern Ontario, summer rain is expected to occur as more frequent extreme rainfall events. Higher temperatures and drought conditions, followed by heavy rainfall, can provide ideal conditions for increasing mosquito abundance and reducing predator populations [87]. If combined with sufficiently extended periods of hot weather to support parasite development, these conditions could further increase transmission potential. Locally transmitted cases of malaria in Suffolk, New York, for example, occurred after heavy rainfall during a particularly hot and dry summer in 1999 [95]. This scenario is less likely in southern British Columbia, where increased rainfall is more likely to occur in the winter rather than the summer [88, 89].

Climate change could be expected, therefore, to increase the potential for locally transmitted, autochthonous malaria in Canada. Climate changes, however, are only one of several determinants of malaria transmission. Endemic malaria transmission occurred in Canada during the 1800s, for example, when temperatures were cooler than today [96]. It is, however, the *combination* of changes in multiple transmission determinants of *sporadic* autochthonous malaria that is of interest.

## 6. A Systems Approach to Malaria in Canada

**Figure 4:**
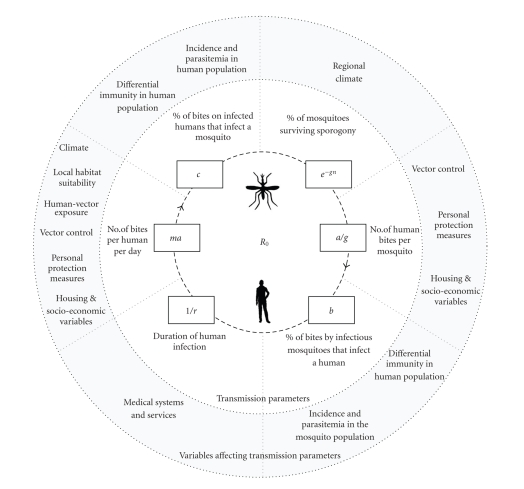
Malaria life cycle model. The inner model of malaria transmission parameters is based on a diagram and parameters from Smith et al. 2007 [97]. Parameter definitions: *R*_0_ (basic reproductive number) = ma^2^ bce^−*gn*^/rg, *a* (human feeding rate): the number of bites on a human, per mosquito, per day, *b* (transmission efficiency): the probability that a human becomes infected from a bite by an infectious mosquito, *c* (transmission efficiency): the probability that a mosquito becomes infected from a bite on an infected human, *g* (death rate of mosquitoes): expected lifespan of a mosquito in days: 1/g, *m*: ratio of mosquitoes to humans, *n* (incubation period): number of days required for the parasite to develop within the mosquito, 1/*r*: duration of infection in humans.

Drawing on the guidelines outlined in Table 1, we can characterize and assess the role of indirect climate impact and nonclimatic determinants of transmission, as well as their interactions. Systems frameworks employ a variety of conceptual tools for system characterization. Here, we adapt the life cycle model of malaria to include the broader determinants of transmission (Figure 4). The basic reproductive number (*R*_0_) of malaria is determined by the biting rates of mosquitoes, and by infection of mosquitoes and humans, which are constrained by the life cycle of mosquitoes and parasites [97]. *R*_0_ represents the transmission potential of a disease, that is to say, the number of secondary cases expected to arise from a primary case in a naïve population [97]. Empirical malaria modeling is often restricted to quantification of these inner parameters and their proximate determinants. The magnitude of these transmission parameters, however, is influenced by a range of mediating variables (outer circle), many of which vary at regional/ local scales or are related to sociopolitical rather than biological systems. As we illustrate, climatic determinants represent only one set of variables affecting malaria transmission parameters.

Of the variables shown in Figure 4, incidence and parasitemia in the mosquito population are perhaps the most likely to experience temporal variation in Canada. Given the low incidence of malaria and current absence of local transmission in Canada, variations in vector control, personal protection measures, housing, socioeconomic variables, differential immunity, and local habitat are unlikely to notably influence transmission. Delays in diagnosis and treatment of malaria in Canada are already a concern [59, 98] and would prolong duration of infection as well as increase transmission potential under conditions of local transmission.

Climate change may have indirect effects on Canadian malaria incidence by affecting the nonclimatic determinants of transmission. For example, any increased incidence of malaria in countries to or from which Canadians travel would affect the magnitude of imported cases and the risk of introduction of parasites into Canadian mosquito populations. This would affect incidence and parasitemia levels in the human population, as well as transmission efficiency (parameters *b* and *c* in Figure 4). Indirect climate change impacts on health vulnerability, socioeconomic status, reduced resources available for vector control measures or health systems, as well as trends in population growth, other diseases, overall poverty and health, and drug use or resistance [80–82, 99], though difficult to quantify, are potentially significant. Similarly, changes in climate such as warmer and longer summers could result in behavioural shifts—such as increased or extended seasonal use of parks and backyard BBQs—that could indirectly affect human-vector exposure and biting rates. Air-conditioning use in southern Canada, which can be expected to increase with warmer summer temperatures, may provide a protective effect, reducing transmission by limiting human exposure to vectors [100].

Increasing immigration and increasing international travel [58, 60, 101, 102]—particularly Canadian immigrants returning to visit friends and relatives in malaria-endemic countries—may increase the likelihood of imported cases, as well as the potential for introduction of parasites into the Canadian mosquito population. The proportion of Canadian immigrants originating from malarial areas has increased significantly in the past 50 years [103] and more Canadians are traveling more often to malarial destinations in Africa, Asia, and South America [104]. Resulting increases in infected and infectious individuals in Canada may increase the potential for transmission of parasites to the local mosquito population as well as potential occurrence of transfusion-transmitted malaria [105]. Canada has, in fact, already recorded malaria cases that may be associated with climate, immigration, and international travel. A dramatic increase in imported *P. vivax* cases in 1995–97 was likely attributable to Canadians of Indian origin who visited the Punjab region of India during a *P. vivax* outbreak associated with higher than normal temperatures and precipitation during a strong El Niño year [59, 64, 65, 106–108]. This example demonstrates the potential for climate impact outside Canada to affect malaria incidence in Canada. Increased incidence and parasitemia in the human population is likely to be most pronounced in urban areas, where travel transit and immigration are the highest.

The on-going, and sometimes rapid, emergence of parasite resistance to antimalarials also has the potential to affect Canadian malaria incidence. Drug resistance has resulted in the emergence and re-emergence of malaria around the world, and it considered to be one of the greatest challenges to global malaria control today [109]. Antimalarial resistance can increase the incidence, severity, and cost of travel-related malaria in Canada by decreasing the efficacy of traveler prophylaxis, complicating selection of prophylaxis and treatment regimes, and increasing the potential for treatment failure. A summary of key trends in the determinants of malaria incidence in Canada is provided in Table 3.

## 7. Conclusion

Our characterization and assessment of the changing climatic and nonclimatic determinants of malaria indicates that Canada may experience increasing imported incidence as well as the potential for emergence of sporadic cases of autochthonous malaria. Our analysis provides a *qualitative* and exploratory assessment of potential climate impacts within the context of other Canadian disease determinants and trends. Whether these trends and parameters would be *quantitatively* sufficient to tip the balance towards sporadic autochthonous transmission is unknown and requires further study.

While well within the capacity of Canada's health system to address, the potential for changes in malaria incidence would require targeted and strategic shifts in health service programming, physician/technologist education and training, travel agent education and travel clinic referral, education of the public, and surveillance. Targeted research to quantify the sensitivity of potential local mosquito transmission to key parameter changes would require the development of process-based models or equivalent empirical models to simulate sporadic incidence. This is consistent with global research recommendations identifying the need for further research on malaria modeling at the regional or national scales and integrating regional environmental and socioeconomic variation [9].

While not quantitatively sufficient to project incidence increase, the results of this review and characterization qualitatively support the merit of targeted regional research to quantify projected transmission potential in Canada. Such an endeavour would be of importance to public health in Canada, but is also of relevance to broader questions related to the impact of climate change on infectious disease occurrence. This review, while regional, highlights the utility of systems frameworks in characterizing potential health risks not readily identified or addressed by global models or climate attribution modeling.

## Figures and Tables

**Figure 1:**
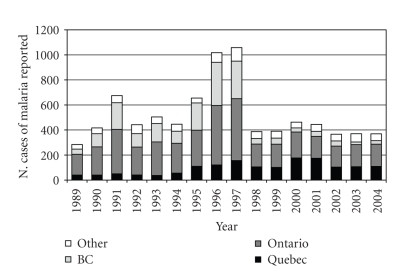
Annual incidence of malaria (caused by all *Plasmodium* species) in Canada. Data obtained from the Public Health Agency of Canada [29].

**Table 1:** Common factors in systems approaches to environment and health.

1. Explicit integration or consideration of the following in analyses:
* *(i) Multiple disciplinary perspectives (e.g., human and biophysical environments)
* *(ii) Nonproximal or qualitative factors affecting transmission (e.g., technology, economic development, public health measures)
* *(iii) Processes acting within, and across, multiple spatial, and temporal scales
* *(iv) Interactions, synergisms, and nonlinearity
2. Use of concept maps (or visual systems graphics) to frame and guide analyses

**Table 2:** *Anopheles* species of Canada and their potential competence as malarial vectors.

Mosquito species	Distribution in Canada	Vector competence as a potential reservoir for *Plasmodium* species
*An. freeborni*	British Columbia (BC)	Competent vector, particularly for *P. vivax,* believed to be the dominant vector of cases in the western USA

*An. quadrimaculatus*	Ontario (ON)	Competent vector for *P. vivax* and *P. falciparum*, believed to be the dominant vector of cases in the eastern USA
	Quebec (QC)	

*An. punctipennis*	British Columbia (BC)	Competent vector for *P. vivax* and *P. falciparum*, but not believed to be dominant vector for human incidence in the USA, possibly due to minimal preference for modern indoor environments
	Manitoba (MB)	
	New Brunswick (NB)	
	Nova Scotia (NS)	
	Ontario (ON)	
	Quebec (QC)	

*An. walkeri*	Manitoba (MB)	Vector competency doubtful; species believed to be of negligible or no importance as a vector of *Plasmodium spp.*
	New Brunswick (NB)	
	Nova Scotia (NS)	
	Ontario (ON)	
	Quebec (QC)	
	Saskatchewan (SK)	

*An. barberi*	Ontario (ON)	Competent vector of *Plasmodium* species, though considered to be of doubtful importance due to its limited contact with man
	Quebec (QC)	

*An. earlei*	All provinces & territories except Newfoundland and Labrador (NF)	Not known to be a competent vector

Sources: [31, 35–37].

**Table 3:** Trends in the determinants of malaria incidence and transmission in Canada.

Driving factors	Potential effect on determinants of malaria transmission	Impact on malaria risk in Canada	Impact on malaria transmission parameters (Figure 4)
Climate change	Improved mosquito habitat in Canada, increased vector populations	Increased probability of local transmission	↑ *ma*
			↓ *g* (↑ *e* ^−*gn*^)
	Extension of the annual period available for parasite replication in mosquitoes in Canada	Increased probability of local transmission	↑ *ma*
			↓ *g* (↑ *e* ^−*gn*^)
	Changes in the distribution and/or incidence in countries outside of Canada to/from which Canadians travel	Impact on the number of imported cases	↑ or ↓ *b*
		Probability of local transmission uncertain	↑ or ↓ *c*

Increasing immigration	Increased introduction of infected individuals	Increased number of imported cases	↑ *b*
		Increased probability of local transmission	↑ *c*

Increasing international travel	Increased introduction of infected individuals	Increased number of imported cases	↑ *b*
		Increased probability of local transmission	↑ *c*

Drug resistance	Decreased efficacy of prophylaxis	Increased incidence and mortality	↑ 1/*r*

Delayed diagnosis	Increased gametocyte incidence	Increased probability of local transmission	↑ 1/*r*
